# Parathyroid carcinoma: from diagnostic challenges to surgical management and long-term outcomes: A single-center retrospective analysis

**DOI:** 10.1097/MD.0000000000049388

**Published:** 2026-06-19

**Authors:** Mustafa Aydemir, Nusret Yilmaz, Ramazan Sari, Cumhur Arici

**Affiliations:** a Division of Endocrinology and Metabolism, Department of Internal Medicine, Akdeniz University School of Medicine, Antalya, Turkey; b Department of Endocrine Surgery, Akdeniz University Faculty of Medicine, Antalya, Turkey.

**Keywords:** hypercalcemia, parathyroid carcinoma, prognosis, recurrence, single-center experience, surgical management

## Abstract

Parathyroid carcinoma (PC) is a rare malignant tumor, accounting for <1% of all cases of primary hyperparathyroidism. It is typically characterized by markedly elevated parathyroid hormone (PTH) levels, severe hypercalcemia, and target organ damage. This study presents a single-center experience in the management of patients with PC and provides a review of the relevant literature. The clinical and laboratory data of 8 patients diagnosed with PC based on pathological examination between September 2008 and February 2024 at the Endocrinology Clinic of Akdeniz University Hospital (Antalya, Turkey) were retrospectively reviewed. Eight patients (3 males) were identified, with a median age of 49 years at diagnosis (range: 46–71 years) and a median follow-up period of 93 months. All patients presented with hypercalcemia, with a median serum calcium level of 13.39 mg/dL (range: 11.2–19) and a median PTH level of 690 ng/L (range: 105–1625). Organ involvement related to hyperparathyroidism was common, most frequently affecting the kidneys (n = 3) and bones (n = 3). All patients underwent en bloc surgical resection, and 2 received adjuvant radiotherapy. Disease recurrence occurred in 3 patients (37.5%) after a median of 24 months following surgery. The cohort size was insufficient to reliably evaluate predictors of recurrence. The 5-year overall survival and disease-specific survival rates were both 87.5%. PC is characterized by a high recurrence rate and limited treatment options beyond complete surgical excision. The diagnosis should be considered in patients presenting with markedly elevated PTH and calcium levels accompanied by a neck mass. En bloc resection remains the mainstay of treatment. The role of adjuvant radiotherapy remains uncertain and warrants further investigation in larger cohorts.

## 1. Introduction

Primary hyperparathyroidism (PHPT) is a prevalent endocrine disorder, with an estimated incidence of 21 cases per 100,000 patient-years. It predominantly affects women in their sixth decade of life.^[1]^ This condition is characterized by elevated serum levels of parathyroid hormone (PTH), which lead to hypercalcemia and hypophosphatemia. These biochemical changes can result in significant organ damage, including urolithiasis, chronic kidney disease, and osteoporosis. In severe cases, hypercalcemia can cause a range of complications, such as altered mental status, nephrogenic diabetes insipidus, dehydration, peptic ulcer disease, pancreatitis, and cataracts.

The vast majority of PHPT cases, approximately 99%, are linked to benign conditions. Most cases are caused by a single PTH secreting adenoma (about 80%) or multiglandular disease (about 15%), which can manifest as multiple adenomas or hyperplasia.^[2,3]^

Parathyroid carcinoma (PC) is an uncommon cause of PHPT, with a frequency ranging from 0.3% to 5%.^[4–6]^ It accounts for only 0.005% of all cancers.^[7]^ Unlike benign parathyroid tumors, PC is typically diagnosed 1 decade earlier, in the fifth decade of life, and is equally distributed between males and females.^[6,8]^ While most cases are functional, secreting PTH, <10% are reported as nonfunctional.^[2,9–12]^

Patients with PC are more likely to present with severe symptoms, larger tumors, and evidence of significant bone and kidney disease. They also exhibit marked hypercalcemia and significantly higher serum PTH concentrations often 5 to 10 times the normal level compared to patients with parathyroid adenoma.^[2,13]^

PC can be sporadic or part of a genetic syndrome, such as hyperparathyroidism-jaw tumor syndrome (HPT-JT) or multiple endocrine neoplasia type 1. HPT-JT is notably linked to mutations in the cell division cycle 73/hyperparathyroidism 2 (*CDC73*/*HRPT2*) gene, while familial isolated hyperparathyroidism involves mutations in both the *CDC73*/*HRPT2* and *GCM2* genes. In rare instances, PC may also occur in the context of *MEN1* (associated with the *MEN1* gene) and *MEN2A* (associated with the *RET* gene).^[2,9–12]^ Aside from these genetic syndromes, no other disease-specific risk factors have been established, although cases have been reported in patients with a history of neck irradiation or end-stage renal disease.^[2]^

Preoperative diagnosis of PC remains challenging, particularly through laboratory parameters. While higher levels of PTH and calcium (Ca) are often more indicative of PC than of adenomas, a definitive diagnosis is most often confirmed postoperatively through histopathology. Pathological signs indicative of malignancy include the presence of fibrous trabeculae, mitotic figures, capsular invasion, and vascular invasion.^[14]^

The primary treatment for PC is en bloc resection, which involves the removal of the affected parathyroid gland along with the ipsilateral hemithyroidectomy. A central lymph node dissection is performed if there are any suspicious findings.^[9]^ Despite aggressive surgical management, PC can recur, with reported recurrence rates ranging from 33% to 82% within 5 years of the initial surgery.^[8,10,11,15]^

Currently, there are no reliable preoperative clinical, biochemical, or radiological indicators that can accurately diagnose malignant parathyroid disease or assess its metastatic potential and prognosis, except in cases with confirmed metastatic disease.^[2]^ Although the 5-year survival rate is a relatively high 85%, disease recurrence is common in the long term, affecting two-thirds of patients up to 20 years after diagnosis.^[2]^ Therefore, further research is essential to identify dependable predictors of malignancy that could help anticipate more complicated clinical courses and enhance patient management.

Given the rarity and complexity of PC, it is crucial to comprehensively examine all aspects of the disease from diagnostic challenges to surgical management, as well as long-term recurrence and survival outcomes. In this study, we aimed to provide valuable insights into the clinical course, treatment modalities, and long-term outcomes of PC by presenting one of the longer follow-up single-center series of patients who were followed at our institution. We believe that our findings will contribute meaningfully to the existing body of literature on the management of PC, helping to guide treatment strategies and improve understanding of the prognosis for patients affected by this rare malignancy.

## 2. Materials and methods

### 2.1. Study design and setting

This study retrospectively reviewed the clinical and laboratory data of 8 patients pathologically diagnosed with PC between September 2008 and February 2024 at the Endocrinology Clinic of Akdeniz University Hospital, Antalya, Turkey. Only patients with a minimum follow-up period of 2 years after diagnosis were included.

### 2.2. Study population and data collection

A total of 8 patients met the inclusion criteria. Clinical characteristics, demographic parameters (age and sex), medical history, and presenting symptoms were recorded. Laboratory data included pre and postoperative serum PTH, Ca, phosphorus (P), 24-hour urinary calcium, 25-hydroxy vitamin D, alkaline phosphatase (ALP), creatinine, and albumin.

Imaging studies comprised ^99m^Tc sestamibi scintigraphy, cervical ultrasonography, and ^18^F-FDG positron emission tomography/computed tomography (CT). Pathological characteristics were retrieved from medical records.

### 2.3. Diagnosis of PC criteria

The diagnosis of PC was established based on histopathological evidence of vascular invasion, perineural invasion, capsular invasion with extension into surrounding tissues, and/or metastasis, in accordance with the fourth edition of the World Health Organization classification of endocrine tumors. One patient underwent primary surgery at an external institution; however, all histological specimens were reevaluated and confirmed by the pathology team at our center.

### 2.4. Statistical analysis

Statistical analyses were performed using Statistical Package for the Social Sciences software (version 26.0; IBM Corp.). Data distribution was assessed with the Shapiro–Wilk normality test.

- Continuous variables were expressed as mean ± standard deviation for normally distributed data or as median with interquartile range (IQR) for non-normally distributed data.
- Categorical variables were presented as frequencies and percentages.

Comparisons between groups were made using the Student *t* test for normally distributed continuous variables and the Mann–Whitney *U* test for non-normally distributed variables. Categorical variables were analyzed with the chi-square test or Fisher exact test, depending on expected cell counts. Given the small sample size (n = 8), only descriptive statistics were used; inferential testing and logistic regression were not performed, as the cohort is insufficiently powered for such analyses.

Specifically:

- Categorical variables (sex, renal involvement, bone involvement, en bloc resection, *CDC73/HRPT2* mutation) were analyzed with the chi-square or Fisher exact test.
- Continuous variables (age at diagnosis, preoperative Ca, PTH, creatinine, ALP, and maximum tumor diameter) were compared using the independent samples *t* test for normally distributed data or the Mann–Whitney *U* test otherwise.

Due to the small sample size, Fisher exact test and Mann–Whitney *U* test were applied for descriptive group comparisons only. Logistic regression analysis was not performed, as the cohort size was insufficient to yield reliable inferential estimates. A *P*-value < .05 was considered statistically significant.

### 2.5. Ethical considerations

All procedures were conducted in accordance with institutional protocols regarding patient data confidentiality and publication ethics.

## 3. Results

### 3.1. Patient characteristics

Among 655 cases of hyperparathyroidism, we identified 8 (1.2%) as PC. The study group included 5 females and 3 males, resulting in a female-to-male ratio of 5:3 (71.4% female). The median age at diagnosis was 49.88 years (range: 46–71). The median follow-up duration was 93 months (IQR = 24–192). The mean calcium level at diagnosis was 13.39 mg/dL (range: 11.2–19), and the mean PTH level was 690.13 ng/L (IQR = 105–1625).

#### 3.1.1. Surgical and postoperative management

The diagnosis of PC was pathologically confirmed after parathyroidectomy in 7 of the patients. These individuals underwent a second surgery, either a subtotal thyroidectomy or lobectomy, within 3 months of their initial operation. The remaining patient underwent a synchronous hemithyroidectomy during the first procedure. Three patients required reoperation due to persistently elevated PTH levels 2 years after their initial surgery, with pathology revealing PC.

To mitigate the risk of hungry bone syndrome (HBS), all patients received preoperative vitamin D supplementation. Calcitriol treatment was initiated in the early postoperative period upon achieving normocalcemia. One patient was also given zoledronic acid preoperatively for concurrent osteoporosis. Despite these measures, 1 patient did develop HBS.

#### 3.1.2. Pathological characteristics

The average tumor size was 27 mm (IQR = 15–50). Pathological examination revealed signs of malignancy, including capsular invasion in 4 patients (50.0%), vascular invasion in 7 patients (87.5%), and intrathyroidal spread in 2 patients (25.0%). These findings, along with key demographic and laboratory parameters, are summarized in Table 1.

**Table 1:** Demographic, clinical, and pathological characteristics of patients with parathyroid carcinoma (n = 8).

Parameter	Value
Average age at diagnosis, yr (median, IQR)	49.88 (46–71)
Average follow-up time, mo (median, IQR)	93 (24–192)
Calcium level at diagnosis, mg/dL (median, IQR)	13.39 (11.2–19)
PTH level at diagnosis, ng/L (median, IQR)	690.13 (105–1625)
ALP level at diagnosis, U/L (median, IQR)	180.50 (76–366)
Nephrolithiasis, n (%)	3 (37.5%)
Brown tumor, n (%)	1 (12.5%)
Parathyroid adenoma localization, n (%)	Right inferior: 7 (87.5%)
	Left inferior: 1 (12.5%)
Average tumor size, mm (median, IQR)	27.0 (15–50)
Capsular invasion, n (%)	4 (50.0%)
Vascular invasion, n (%)	7 (87.5%)
Intrathyroidal extension, n (%)	2 (25.0%)
Postoperative calcium level, mg/dL (median, IQR)	9.58 (8.65–10.8)
Postoperative PTH level, ng/L (median, IQR)	64.25 (13–140)

ALP = alkaline phosphatase, IQR = interquartile range, PTH = parathyroid hormone.

The mean postoperative calcium level was 9.58 mg/dL (range: 8.65–10.8), and the mean postoperative PTH level was 64.25 ng/L (range: 13–140).

### 3.2. Clinical presentation of patients with PC

Seven of the 8 patients in our cohort initially presented with constitutional symptoms commonly associated with advanced hyperparathyroidism, including fatigue, bone pain, arthralgia, and muscular pain. In contrast to the often-asymptomatic nature of benign PHPT, all patients in this study exhibited at least 1 clinical symptom, with several experiencing a constellation of symptoms.

One patient presented with severe hypercalcemia, with a serum calcium level of 19 mg/dL, while the remaining 7 had moderate hypercalcemia. Other notable clinical findings included a significant weight loss of 5 kg over 1 month in 1 patient, memory loss in another, and a palpable neck mass in 3 patients. The detailed clinical characteristics of all 8 patients are summarized in Table 2.

**Table 2:** Presenting symptoms in 8 patients with parathyroid carcinoma.

Symptom	1	2	3	4	5	6	7	8	No. of patients
Asymptomatic									0/8 (0%)
Fatigue	+	+	+	+	+	+	+		7/8 (87.5%)
Bone pain	+	+	+	+	+	+		+	7/8 (87.5%)
Headaches		+	+						2/8 (25%)
Joint pain	+	+	+	+	+	+		+	7/8 (87.5%)
Weight loss							+5 kg		1/8 (12.5%)
Dyspepsia	+		+	+			+	+	5/8 (62.5%)
Muscular pain	+	+	+	+	+	+		+	7/8 (87.5%)
Memory deficit							+		1/8 (12.5%)
Constipation		+					+		2/8 (25%)
Paresthesias			+	+	+				3/8 (37.5%)
Polyuria		+			+		+	+	4/8 (50%)
Polydipsia		+			+		+	+	4/8 (50%)
Neck mass	+		+				+		3/8 (37.5%)
Neck pain			+	+		+			3/8 (37.5%)

#### 3.2.1. Demographics, clinical presentation, and outcomes

The cohort consisted of 3 males and 5 females. The ages of the patients ranged from 46 to 71 years. One patient presented with severe hypercalcemia (calcium level of 19 mg/dL) and a concurrent memory deficit. The memory impairment resolved following the medical management of hypercalcemia, and the patient proceeded to surgery once their calcium levels were normalized. PTH levels at admission ranged from a low of 105 ng/L to a high of 1625 ng/L.

#### 3.2.2. Renal and imaging findings

Three patients presented with acute renal failure at the time of diagnosis. Unfortunately, their renal function did not improve in the postoperative period and subsequently progressed to chronic renal failure. Preoperative imaging, including parathyroid ultrasonography and scintigraphy, successfully localized the PCs in all patients. Seven of the tumors were located in the right inferior parathyroid gland, and 1 was found in the left inferior gland.

#### 3.2.3. Postoperative status

Postoperatively, 1 patient continued to exhibit elevated calcium levels, indicating persistent disease. The presentation, demographic characteristics, and laboratory findings for all patients are summarized in Table 3.

**Table 3:** Presentation, demographics, and laboratory results of the patients.

Patients	1	2	3	4	5	6	7	8
Age	57	55	51	71	65	57	59	46
Sex	M	F	F	F	M	F	M	F
Laboratory results at diagnosis								
Calcium (mg/dL)	14.24	13.09	12.06	11.2	13.2	12.5	19	11.8
Phosphorous (mg/dL)	1	4	1.6	3	2.1	2.9	1.1	1.4
PTH (ng/L)	1625	914	250	105	1067	228	874	458
25(OH)Vit D (μg/L)	21	13.9	11	22	14	17	7.8	6.5
Albumin (g/L)	3.8	3.8	4.5	4.4	4.3	4.8	3.1	4.2
ALP (U/L)	366	298	120	76	160	158	110	166
Creatinine (mg/dL)	1.2	4.1	0.75	0.88	0.97	0.58	1.52	0.41
24-h urine calcium (mg/d)	410	400	347	350	1270	450	549	620
Tumor localization	R.I.	R.I.	R.I.	R.I.	R.I.	L.I.	R.I.	R.I.
Laboratory results at postoperative stage								
Calcium (mg/dL)	9.13	8.65	9.96	10.8	9.7	9.45	9.2	9.71
Phosphorous (mg/dL)	2.6	4.9	1.8	2.1	2.4	2.71	3.05	1.45
PTH (ng/L)	34	42	93	78	58	13	140	56
25(OH)Vit D (μg/L)	41	24	14	31	22	17	19	20
Albumin (g/L)	4	4	3.7	4.7	4	4.2	4.3	1.2
ALP (U/L)	104	94	461	69	53	150	140	140
Creatinine (mg/dL)	1.1	4.4	0.48	0.84	0.74	0.56	1.1	0.63
24-h urine calcium (mg/d)	185	277	402	150	100	150	195	437

ALP = alkaline phosphatase (30–120 U/L); calcium (8.8–10.6 mg/dL); phosphorous (2.4–4.4 mg/dL), F = female, L.I. = left inferior, M = male, PTH = parathyroid hormone (15–65 ng/L); 25(OH) vitamin D (30–100 μg/L); 24-h urine calcium (0–300 mg/d), R.I. = right inferior.

### 3.3. Complications of PHPT

Hypercalcemia-mediated target organ damage was a common finding among the 8 patients in our cohort. A majority of patients (n = 8) exhibited at least 1 complication, with hypertension observed in 6 patients, renal failure in 3, and peptic ulcer disease in 4. In addition, 1 patient presented with a brown tumor, a classic manifestation of severe hyperparathyroidism.

#### 3.3.1. Skeletal and renal complications

Renal ultrasound was performed on all 8 patients preoperatively, revealing nephrolithiasis (kidney stones) in 3 of them. Furthermore, dual-energy X-ray absorptiometry scans, which were available for all patients, showed skeletal complications. Specifically, osteoporosis was diagnosed in the femoral neck of 1 patient and in the lumbar spine of 2 patients. Notably, none of the patients had a history of prior osteoporotic fractures. The full spectrum of complications of PHPT in our cohort is summarized in Table 4.

**Table 4:** Complications of primary hyperparathyroidism.

Complication	Patients with available data (n)	Patients with complications, n (%)
Brown tumor	8	1 (12.5%)
Nephrolithiasis	8	3 (37.5%)
Renal failure	8	3 (37.5%)
Osteoporosis	8	3 (37.5%)
Femoral neck osteoporosis	8	1 (12.5%)
Lumbar spine osteoporosis	8	2 (25.0%)
Distal radius osteoporosis	8	1 (12.5%)
Hypertension	8	6 (75%)
Peptic ulcer disease	8	4 (50.0%)

### 3.4. Pathology reports

Eight patients underwent an en bloc parathyroid tumor resection, with 3 of them also having a total thyroidectomy. The pathological findings were diverse (Table 5), but key features of malignancy were prevalent. The majority of cases showed capsular invasion (n = 4), vascular invasion (n = 7), and parathyroid capsule invasion (n = 4).

**Table 5:** Pathology reports.

	1	2	3	4	5	6	7	8
Tumor diameter (mm)	40	20	50	18	20	23	30	15
Necrosis				+	+			
Mitotic activity (BBA)		5		5	5		5	5
Calcification					+			
Lymphatic invasion		+	+	+				
Vascular invasion	+	+	+	+		+	+	+
Tumor capsule invasion	+	+	+	+			+	+
Parathyroid capsule invasion	+	+	+	+			+	+
Soft tissue invasion			+	+				
Thyroid capsule invasion		+	+	+		+		
Surgical margin	R.I.	R.I.	R.I.	R.I.	R.I.	L.I.	R.I.	R.I.
p53 (%)								
Ki-67 (%)		6	5	5	5	2–3	5–10	4
Positive lymph node			+					+
Extraglanduler invasion			+	+				

L.I. = left inferior, R.I. = right inferior.

Furthermore, 2 patients were found to have lymph node metastasis, and 2 had extraglandular invasion. The average tumor diameter was 27 mm, with a range from 15 mm to 50 mm. The mitotic index was evaluated in 5 patients, but no specific values were reported.

#### 3.4.1. Postoperative outcomes and genetic analysis

One patient (12.5%) developed HBS in the postoperative period. In addition, 1 patient (12.5%) experienced transient hypoparathyroidism. Genetic analysis for germline *CDC73*/*HRPT2* mutations was performed, and 2 of the 8 patients (25%) tested positive. All these findings are summarized in detail in Table 5.

### 3.5. Treatments for PCs

All 8 patients underwent en bloc parathyroid tumor resection as their primary treatment. In addition, 3 of these patients had a total thyroidectomy. Despite these surgical interventions, 3 patients (Patients 3, 4, and 8) required reoperation due to persistently elevated calcium and PTH levels during their postoperative follow-up, indicating disease recurrence.

#### 3.5.1. Management of recurrent disease

The management of recurrent disease varied among the patients.

Patient 3 experienced consistently high calcium levels, prompting a positron emission tomography-CT scan that revealed a focus in the lung, necessitating a third surgical intervention. Due to continued hypercalcemia, calcimimetic therapy and weekly zoledronic acid were initiated. When calcium levels remained dangerously high, intermittent hemodialysis was performed to reduce them.

Patient 4 underwent radiotherapy and chemotherapy following a second operation due to local invasion.

Patient 8 had a second operation 2 years after her initial surgery, during which lymph node metastasis was detected. She subsequently underwent a total of 4 operations over a span of 2 to 3 years due to the development of local nodes. Remarkably, this patient remains alive 192 months after her initial diagnosis, highlighting the long-term management required for recurrent PC. The full details of the treatments received by all patients are summarized in Table 6.

**Table 6:** Treatments for parathyroid carcinomas.

	1	2	3	4	5	6	7	8
Total operation	+	+	+	+	+	+	+	+
Recurrence surgery			++	+				+++
Radiotherapy			+	+				
Chemotherapy				+				
Calcimimetic			+					
Biphosphonate			+	+		+		
Calcitriol		+				+		
Oral calcium		+				+		
Levothyroxine		+	+	+				

### 3.6. Recurrence and mortality

#### 3.6.1. Long-term follow-up and outcomes

The follow-up period for the patients ranged from 24 to 192 months, with a median of 93 months. Disease remission, defined as the absence of structural or biochemical evidence of disease, was achieved in 5 patients (62.5%) during this follow-up period (Table 7).

**Table 7:** Laboratory data at last evaluation.

Patient/sex	Calcium (mg/dL)	Phosphorus (mg/dL)	24-h urinary calcium (mg/d)	Alkaline phosphatase (U/L)	PTH (ng/L)	Creatinine (mg/dL)	25(OH)D (ng/mL)	Follow-up (mo)	Recurrence	Death
Normal range	8.4–10.2	2.3–4.7	<250	40–150	15–65	0.4–1.2	8–56			
1/M	9.1	2.6	185	104	34	1.1	41	84	No	No
2/F	8.6	4.9	277	94	42	4.4	24	24	No	No
3/F	12.5	1.8	402	461	1677	0.4	14	144	BED + SED	Yes
4/F	10.8	2.1	150	69	108	0.8	31	60	BED + SED	Yes
5/M	9.7	2.4	100	53	58	0.7	22	84	No	No
6/F	9.4	2.7	150	150	13	0.5	17	60	No	No
7/M	9.2	3.0	195	140	30.9	1.1	19	96	No	No
8/F	11.3	1.4	437	140	181	0.6	20	192	BED + SED	No

BED = biochemical evidence of disease, F = female, M = male, NA = not available, PTHi = intact parathyroid hormone, SED = structural evidence of disease.

#### 3.6.2. Disease recurrence and management

Recurrence was observed in 3 patients (37.5%) after a median of 24 months (range: 12–36) following their initial surgery. Biochemical recurrence was noted in all 3 patients, while structural evidence of disease was present in 2 of them. Specifically, Patient 3 had locoregional disease, and Patient 4 had distant metastasis to the lung.

Two patients (Patients 3 and 4) received adjuvant radiotherapy, and Patient 4 also underwent adjuvant chemotherapy. Despite these treatments, both patients ultimately succumbed to their disease. Patient 3 died 144 months after diagnosis due to hypercalcemia, and Patient 4 died 60 months after diagnosis, also from hypercalcemia-related complications.

In contrast, Patient 8 had a remarkable clinical course. She underwent a second operation 2 years after her initial surgery, at which time lymph node metastasis was detected. She subsequently underwent a total of 4 operations over 2 to 3 year intervals to manage recurrent local nodes. Despite these challenges, she remains alive and well 192 months after her initial diagnosis. The most recent postoperative laboratory data for the cohort is presented in Table 7.

#### 3.6.3. Factors associated with disease recurrence

Upon comparing patient characteristics based on disease recurrence, we found no significant differences across the analyzed variables. None of the examined features – including age at diagnosis, preoperative levels of calcium, PTH, creatinine, and ALP, tumor size, sex, renal and bone manifestations, en bloc resection, and the presence of a *CDC73*/*HRPT2* mutation – showed a statistically significant association with the development of recurrence (Table 8).

**Table 8:** Clinical findings in patients with and without recurrence.

Clinical feature	PC with recurrence (n = 3)	PC without recurrence (n = 5)	*P* value
Age at diagnosis (mean, yr)	56	58.6	1.000
Female (n)	3	2	1.000
Male (n)	0	3	1.000
Preoperative calcium (mean, mg/dL)	11.6	14.5	1.000
PTH (mean, pg/mL)	271	941.6	1.000
Creatinine (mg/dL)	0.68	1.65	1.000
Alkaline phosphatase (mean, U/L)	120.6	218.4	.19642
Tumor greater dimension (mean, mm)	27.6	26.6	.19642
Lymph node metastasis (n)	2	0	.10714
Extraglanduler invasion (n)	2	0	.10714
Renal manifestation (n)	0	3	.19642
Bone manifestation (n)	2	1	.46428
En bloc resection (n)	3	5	1.000
*CDC73*/*HRPT2* mutation (n)	2	0	.10714

PC = parathyroid carcinoma, PTH = parathyroid hormone.

#### 3.6.4. Statistical analysis and key findings

We observed no significant differences between the 2 patient subgroups (those with and without recurrence) in terms of sex, mean age at diagnosis, serum calcium, ALP, creatinine, and tumor size. Similarly, the prevalence of renal manifestations (failure or stones) and bone manifestations (pathological fractures, osteoporosis, and brown tumors) did not differ significantly.

A logistic regression analysis further confirmed that demographic characteristics (age, sex), laboratory data (PTH, calcium, and creatinine), tumor features (size), histological features (vascular invasion, capsular invasion, and extraglandular extension), and the specific surgical procedure were not associated with recurrence (*P* > .05). We also did not find a statistically significant difference in recurrence, laboratory data, or renal and bone involvement between patients with and without a *CDC73*/*HRPT2* germline mutation. However, given the small cohort size (n = 8 with only 3 recurrences), inferential testing is underpowered, *P*-values are unstable, and the absence of statistical significance does not imply the absence of clinically meaningful associations. Logistic regression analysis was therefore not performed.

The statistical analyses, which included the Mann–Whitney *U* test for continuous variables and Fisher exact test for categorical variables (due to small sample sizes), were applied for descriptive group comparisons only. Given that the cohort comprised only 8 patients with 3 recurrences, the study is underpowered; p-values are unstable and regression analyses are unreliable. Absence of statistical significance should not be interpreted as absence of a clinically meaningful association. Accordingly, these analyses are presented as descriptive observations only, and no independent predictors of recurrence can be reliably identified from this dataset.

### 3.7. Survival analysis and outcomes

**Figure 1.**
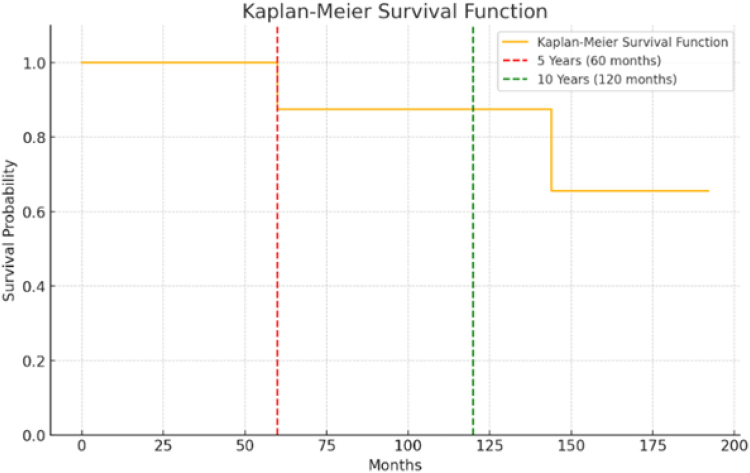
Total survival in patients with parathyroid carcinoma. The Kaplan–Meier survival function graph shows 5- and 10-year survival rates. At the end of 5 years, the survival rate is 87.5%, and it remains the same at the end of 10 years.

Of the 8 patients in our cohort, 2 (25%) died due to severe hypercalcemia associated with advanced malignancy. The remaining 6 patients are currently alive. A Kaplan–Meier survival analysis was performed to assess long-term survival rates. The survival function graph (Fig. 1) illustrates the probability of survival over time. According to this analysis, the 5-year survival rate was 87.5%. This survival probability remained unchanged at the 10-year mark, as indicated by the graph’s plateau. The red dashed line on the graph represents the 5-year (60-month) survival point, and the green dashed line marks the 10-year (120-month) point. These findings provide a clear visual representation of patient outcomes in the long-term management of PC (Fig. 1).

### 3.8. Prognostic factors and survival analysis

In our study, we did not find any statistically significant differences in survival based on sex, age at diagnosis, tumor size, or preoperative calcium level. However, a notable observation was that patients with preoperative serum calcium levels >13.5 mg/dL were significantly younger than those with lower calcium levels (37.8 ± 13.9 years vs 52.6 ± 12.7 years, *P* = .03).

#### 3.8.1. Trends in survival and disease characteristics

While not statistically significant, there was a clear trend toward shorter survival in patients with severe hypercalcemia. Interestingly, all patients with a preoperative serum calcium level below 13.5 mg/dL are still living, suggesting that the degree of hypercalcemia may serve as a potential, albeit nonsignificant, prognostic indicator. A specific trend was observed in male patients, who appeared to have a clinical profile characterized by younger age and more severe hypercalcemia, which seemed to correlate with an increased risk of disease-related mortality.

#### 3.8.2. Genetic and clinical associations

Lastly, our analysis did not reveal any statistically significant differences between patients with or without a *CDC73*/*HRPT2* germline mutation in terms of disease recurrence, laboratory data, or the presence of renal and bone involvement. Based on our limited patient cohort, we concluded that our findings were insufficient to determine whether this specific genetic mutation serves as a significant predictive factor for the clinical outcomes in question. We believe that further research with larger sample sizes is necessary to draw more definitive conclusions.

## 4. Discussion

PC is a rare neuroendocrine malignancy with an estimated annual incidence of 2 cases per 10 million, accounting for approximately 0.005% of all cancers.^[16]^ Diagnosis of PC typically occurs in the fifth decade of life, with a median age of 49.88 years at diagnosis in our study.^[8,17–27]^ Both sporadic and familial cases of PC have been reported. In addition to prior cervical radiation therapy, secondary and tertiary hyperparathyroidism have been suggested as predisposing factors for this neoplasm; however, the evidence supporting these associations is limited and inconsistent.^[2]^

In this retrospective single-center analysis, we evaluated the clinical characteristics, management strategies, and long-term outcomes of 8 PC patients followed at our clinic between September 2008 and February 2024. Over a median follow-up period of 93 months (24–192 months), recurrence was observed in 37.5% of patients, while the 5-year overall survival and disease-specific survival rates were both 87.5%.

### 4.1. Demographic and clinical features

The median age at diagnosis in our study was 49.88 years, which is consistent with the literature reporting a presentation in the fifth decade of life.^[8,17–19]^ Unlike benign parathyroid tumors, PC is reported to show no sex predilection, occurring equally in men and women.^[13]^ However, our cohort observed a slight predominance of female patients (5 women, 3 men, 71.4% female), which is likely attributable to our small sample size.

A majority of our patients (87.5%) presented with constitutional symptoms such as fatigue, bone pain, arthralgia, and muscular pain. In addition, a palpable neck mass was identified in 37.5% (3/8) of patients, a rate consistent with other series reporting neck masses in 31% to 64% of patients, often being the most common finding in hormonally silent PC cases.^[19,28,29]^ Target organ damage from PHPT was notably prevalent in our cohort, with hypertension in 75% (6/8), nephrolithiasis in 37.5% (3/8), renal failure in 37.5% (3/8), and peptic ulcer disease in 50% (4/8) of cases. These findings consistently reflect the aggressive clinical course of PC and are supported by previous publications, such as those by Cunha et al.^[30]^

### 4.2. Biochemical and pathological indicators

While there are no specific laboratory or radiological criteria for PC diagnosis, clinical and biochemical findings in carcinoma cases are generally more severe than in benign PHPT. In our study, the median serum calcium level was 13.39 mg/dL (11.2–19 mg/dL) and the mean PTH level was 690 pg/mL (105–1625 pg/mL). The literature suggests that PC cases typically have median serum calcium levels of 15.9 mg/dL, with approximately 75% of patients exceeding 14 mg/dL and only 10% below 13 mg/dL.^[31,32]^ In our cohort, only 2 patients had calcium levels above 14 mg/dL, while 2 had levels below 12 mg/dL. This is generally consistent with the literature, though our cohort exhibited a slightly lower distribution of extreme hypercalcemia compared to some larger series. ALP levels are reported to be higher in PC compared to parathyroid adenomas, with a threshold of 300 U/L below which carcinoma is considered unlikely.^[32]^ In our study, 1 patient had ALP levels exceeding this threshold. While average PTH levels 5 to 10 times the normal range (i.e., above 500 pg/mL) are considered strong indicators for carcinoma, preoperative laboratory data are often insufficient to reliably predict malignancy.^[33,34]^

Pathologically, a remarkably high vascular invasion rate of 87.5% (7/8) was found in our study. This rate is significantly higher than those reported by Clayman et al (37%)^[35]^ and Fernandez-Ranvier et al (30.8%),^[36]^ but aligns with higher rates reported by Healy et al (62.5%),^[37]^ Akirov et al (87.5%),^[33]^ and Gogas et al (85.7%).^[17]^ Such elevated rates, especially in studies with small sample sizes, may be a contributing factor. Capsular invasion was observed in 50% (4/8) of our patients. Lymph node metastasis and distant metastases are reported at diagnosis in approximately 15% to 30% and 33% of cases, respectively.^[2]^ In our cohort, lymph node metastasis was detected in 25% (2/8) and distant organ metastases (lungs and skin) in 25% (2/8) of patients, consistent with existing literature.^[14,38–40]^ Regarding tumor localization, all our cases (100%) were located in the inferior parathyroid glands, which aligns with the literature indicating a higher tendency for PC to occur in inferior glands.

### 4.3. Surgical management and postoperative course

The primary treatment for PC is en bloc resection of the affected parathyroid gland, along with ipsilateral hemithyroidectomy and central lymph node dissection if suspicious findings are present.^[8–11,15]^ All 8 patients in our study underwent en bloc resection. However, due to difficulties in achieving a definitive intraoperative diagnosis, complete resection is not always feasible.^[6,11]^ In our single-center experience, 3 patients (37.5%) required reoperation due to persistently elevated calcium and PTH levels after initial surgery, underscoring the critical importance of complete resection. Incomplete resection has been associated with recurrence rates up to 50% and disease-related mortality rates reaching 46%.^[6,7]^ Therefore, ensuring “complete resection” during surgical treatment is of vital importance.^[41–43]^

In the postoperative period, PC patients with severe PHPT and high bone turnover are susceptible to developing HBS after parathyroidectomy.^[35]^ In our study, HBS developed in 1 patient (12.5%), while transient hypoparathyroidism was observed in 1 patient (12.5%) and persistent hypoparathyroidism in another (12.5%). These rates are consistent with the literature regarding risk factors (high PTH and ALP levels, evidence of bone disease) and pathogenesis.^[11,44]^

### 4.4. Recurrence and survival outcomes

PC is characterized by a high recurrence rate. Recurrences typically occur within 2 to 3 years after initial surgery, though late recurrences up to 23 years have been reported.^[18,22]^ The median recurrence time in our cohort also aligns with this general pattern.^[22]^ In our study, recurrence was observed in 3 patients (37.5%) within a median of 24 months (12–36 months) following the first surgery. This rate is lower than the 56% reported by Busaidy et al at MD Anderson and 67% reported by Wynne et al at Mayo Clinic, but falls within the broad range of 33% to 82% reported in the general literatüre.^[8]^ Wynne et al documented a 67% recurrence rate in a study of 43 patients followed at Mayo Clinic.^[18]^ Previous cohorts have shown that the average time to first recurrence ranges from 2.5 to 8.4 years.^[45]^ However, much later recurrences have also been documented, and these patients should be monitored for life with serum calcium and PTH levels.^[46]^ Due to the rarity of PC, there is no consensus on follow-up strategies.^[47]^ In cases of recurrence, patients typically present with elevated PTH and calcium levels, although hypercalcemic crisis is very rare.^[7]^ Patients in clinical remission are monitored with serum calcium and PTH measurements every 6 months. In the event of rising PTH and calcium levels indicating recurrence, patients should undergo localized imaging studies, including neck ultrasound, CT, magnetic resonance imaging, sestamibi scanning, or fluorodeoxyglucose-positron emission tomography. Monitoring of nonfunctioning PC should be performed through imaging studies.^[11,47]^ Treatment involves the surgical excision of resectable disease following localization.^[7]^

The cohort size was insufficient to reliably evaluate predictors of recurrence; with only 8 patients and 3 recurrences, the study is underpowered and inferential testing is not meaningful. It is important to remember that recurrent cases often present with elevated PTH and calcium levels, and patients should be closely monitored with serum calcium and PTH levels for life.

The 5-year overall and disease-specific survival rates in our study were both 87.5%. This is consistent with the 5-year survival rate of 86% reported in a review of 286 patients from the American National Cancer Database and 85% in other studies.^[34]^ Furthermore, our study found a 10-year survival rate of 87.5%. This figure is higher than the 10-year survival rates ranging from 49% to 77% reported in the literature. This may reflect the aggressive surgical management, meticulous follow-up, and early interventions performed at an experienced center like Akdeniz University. Two patients died due to severe hypercalcemia associated with advanced malignancy, which aligns with the literature suggesting that metabolic complications are the main cause of disease-related deaths.

### 4.5. Prognostic factors and genetic insights

Some data suggest that *CDC73*/*HRPT2* mutations may be associated with poor prognosis and an increased risk of recurrence.^[48]^ In our study, germline *CDC73*/*HRPT2* mutations were detected in 2 patients (25%), and both experienced recurrence; however, as only 2 of 8 patients underwent *CDC73* testing, this represents an anecdotal observation rather than a meaningful association. No conclusions regarding mutation-associated recurrence risk can be drawn from these data. Further research with larger patient populations and systematic genetic testing is needed to draw definitive conclusions.

### 4.6. Treatment challenges and future directions

Data on the benefits of adjuvant therapies (radiotherapy, chemotherapy) are insufficient, and these treatments are primarily used for palliative purposes. Given PC’s resistance to radiotherapy, its role in management is limited.^[15]^ Standard chemotherapy regimens have shown disappointing results, making surgery the only potentially curative approach when the disease recurs.^[5]^ There is a clear need to discover more effective treatments to address both tumor burden complications and hypercalcemia. Next-generation sequencing methods have significantly expanded our understanding of genomic alterations in PC. Recent studies suggest that genes in the PIK3CA/AKT/mTOR pathway may be potentially targetable in PC.^[46,49]^ Deaths from parathyroid cancer are typically due to metabolic complications or refractory severe hypercalcemia, rather than the primary cancer itself.^[34]^

## 5. Limitations of the study

The main limitation of this study is the small number of patients (n = 8) due to the rarity of this malignancy. This small sample size limited our ability to find statistically significant differences in analyses and prevented us from drawing definitive conclusions for some potential prognostic factors (e.g., the relationship between *CDC73*/*HRPT2* mutations and recurrence). Furthermore, genetic analyses could not be performed on all patients, which restricted a more comprehensive evaluation of genetic predisposition.

## 6. Conclusion

PC is a rare and aggressive malignancy characterized by a high recurrence rate and limited treatment options. Our study supports the notion that PC should be considered in patients presenting with severe hypercalcemia, high PTH levels, and a neck mass. En bloc resection remains the primary treatment method, and achieving complete resection is critical for long-term survival. The role of adjuvant radiotherapy remains uncertain; both patients who received radiotherapy in this series ultimately died of disease, and no definitive conclusions regarding its benefit can be drawn from this small cohort. Due to its rarity, larger-scale, multicenter studies are needed to better understand the disease biology, identify prognostic factors, and develop more effective treatment strategies.

## Author contributions

**Conceptualization:** Mustafa Aydemir.

**Data curation:** Mustafa Aydemir, Nusret Yilmaz, Ramazan Sari.

**Formal analysis:** Mustafa Aydemir, Nusret Yilmaz.

**Funding acquisition:** Mustafa Aydemir.

**Methodology:** Mustafa Aydemir, Ramazan Sari, Cumhur Arici.

**Project administration:** Mustafa Aydemir.

**Validation:** Mustafa Aydemir, Nusret Yilmaz.

**Writing– original draft:** Mustafa Aydemir.

**Writing – review & editing:** Mustafa Aydemir.

**Resources:** Nusret Yilmaz, Cumhur Arici.

**Supervision:** Nusret Yilmaz, Ramazan Sari, Cumhur Arici.

**Investigation:** Cumhur Arici.
